# Acute cholecystitis – early laparoskopic surgery versus antibiotic therapy and delayed elective cholecystectomy: ACDC-study

**DOI:** 10.1186/1745-6215-8-29

**Published:** 2007-10-04

**Authors:** Kilian Weigand, Jörg Köninger, Jens Encke, Markus W Büchler, Wolfgang Stremmel, Carsten N Gutt

**Affiliations:** 1 Department of Gastroenterology and Hepatology, University of Heidelberg, Germany; 2 Department of General Surgery, University of Heidelberg, Germany

## Abstract

**Background:**

Acute cholecystitis occurs frequently in the elderly and in patients with gall stones. Most cases of severe or recurrent cholecystitis eventually require surgery, usually laparoscopic cholecystectomy in the Western World. It is unclear whether an initial, conservative approach with antibiotic and symptomatic therapy followed by delayed elective surgery would result in better morbidity and outcome than immediate surgery. At present, treatment is generally determined by whether the patient first sees a surgeon or a gastroenterologist. We wish to investigate whether both approaches are equivalent. The primary endpoint is the morbidity until day 75 after inclusion into the study.

**Design:**

A multicenter, prospective, randomized non-blinded study to compare treatment outcome, complications and 75-day morbidity in patients with acute cholecystitis randomized to laparoscopic cholecystectomy within 24 hours of symptom onset or antibiotic treatment with moxifloxacin and subsequent elective cholecystectomy. For consistency in both arms moxifloxacin, a fluorquinolone with broad spectrum of activity and high bile concentration is used as antibiotic. Duration: October 2006 – November 2008

**Organisation/Responsibility:**

The trial was planned and is being conducted and analysed by the Departments of Gastroenterology and General Surgery at the University Hospital of Heidelberg according to the ethical, regulatory and scientific principles governing clinical research as set out in the Declaration of Helsinki (1989) and the Good Clinical Practice guideline (GCP).

**Trial Registration:**

ClinicalTrials.gov NCT00447304

## Background

### Medical problem

Acute cholecystitis is one of the most significant acute diseases in the Western World, and may be associated with only mild pain and nausea or become a severe, life-threatening illness due to complications. Acute cholecystitis is mainly caused by gall stones, whilst cholestasis is mainly associated with superinfection with bacteria, in general species of enterobacteria, enterococci, bacteroides and anaerobic streptococci [1].

The principal complication is recurrent biliary colic and cholestasis. The latter may lead to ascending cholangitis, and whilst this can be managed with antibiotics, other complications can not be cured conservatively, such as gangrenous changes, gall bladder perforation and biliary leakage, and acute necrotic gallstone pancreatitis [2-5]. Liver abscesses and underlying incidental carcinoma have also been reported in some cases [2,6].

The risk of developing second and subsequent episodes of acute cholecystitis is higher than the risk of suffering an initial episode [7,8]. Laparoscopic cholecystectomy is therefore usually recommended, but whether this should be performed immediately or after first giving antibiotic treatment to allow the acute condition to subside is controversial [9-12].

### Immediate surgery versus conservative procedure with subsequent elective surgery

The approach taken is often decided by whether the patient first sees a gastroenterologist, who favors conservative initial antibiotic therapy with later elective surgery, or a surgeon, who favors immediate surgery.

It is still unclear which approach is better in medical and health economic terms. The infection may not respond to conservative treatment on one hand, on the other hand surgical intervention while the disease is acute may increase complications, and conversion to open surgery may be necessary. It is unclear whether it is better to conduct early cholecystectomy, thereby avoiding the risk of recurrent cholecystitis or pancreatitis.

A meta-analysis by Papi et al. (2003) including 12 prospective randomized trials showed no significant difference for morbidity and mortality between immediate surgical intervention (laparoscopic or open) and elective surgery after the acute inflammation had subsided [13]. The numbers of patients and rates of complications were too low to enable any conclusions to be drawn. Also, their definition of "immediate" was between 1 and 7 days after disease onset, while the modern standard favors laparoscopic surgery within 24 hours of onset [14,15].

All studies in the meta-analysis had been performed between 1970 and 2000, and Papi concluded that new studies in an adequate number of patients to show statistical significance should be performed [13]. Two subsequent prospective randomized trials concerning the appropriate timing for surgery also failed to lead to conclusive results, except for a slightly shorter hospital stay in patients treated with immediate surgery [16,17].

In many centers early cholecystectomy is well established although the evidence is not yet conclusive. To our knowledge there has never been a study in which both specialties – gastroenterology and surgery – are equally involved.

### Choice of antibiotic

Moxifloxacin (Avalox^®^) covers the spectrum of gram-positive, gram-negative and anaerobic bacteria usually responsible for intra-abdominal infections [18-20]. It can be applied orally or intravenously in a single dose of 400 mg/day, resulting in bile concentrations significantly above the minimum inhibitory concentration [21], and 3–4 times higher than plasma concentrations [22].

A controlled double-blinded study in 379 patients in the USA showed moxifloxacin to be at least as effective as standard treatment by with piperacillin + tazobactam i.v. followed by oral amoxicillin+clavulanate in complicated intra-abdominal infections [23,24]. A European prospective randomized and controlled open-label study showed equivalent efficacy for moxifloxacin and ceftriaxone plus metronidazole (AIDA study) [25]. Moxifloxacin was effective and well-tolerated in both studies, with gastrointestinal disorders like nausea and diarrhea being the most frequent adverse events [26,27].

## Study Design

### Aim of the study

The objective of this trial is to compare the 75-day morbidity of two different approaches to the treatment of acute cholecystitis: (i) laparoscopic cholecystectomy within 24 hours of hospital admission; and (ii) initial antibiotic treatment with moxifloxacin followed by cholecystectomy in the infection-free interval (Day 7 to 45).

### Organization of the study

The trial is a GCP-compliant, multicenter, prospective, randomized non-blinded study. Patients with acute cholecystitis meeting the inclusion criteria are randomized to one of the treatment arms. The study is being audited by members of a contract research organization (CRO) and may be subject to government inspection. The trial was approved by the German authorities and Ethical committees.

### Number of patients needed

The primary aim of the study is to compare morbidity in the two groups 75 days after enrolment. A difference in morbidity of less than 10% is defined as equivalent. The null-hypothesis is /ρ_M1 _- ρ_M2 _/> 0.1, where ρ_Mi _is the morbidity rate of treatment group i. Complications are expected in 16% of patients in each group; each group therefore needs to enroll 273 patients to permit verification of the null-hypothesis with an α-error of 0.05 and a β-error at 0.15, yielding a power of 90%. Assuming a validity rate of 85%, 322 patients are required per group, resulting in a total patient sample of 644.

## Eligibility criteria

### Inclusion criteria

• Age ≥ 18 years

• Patients with acute cholecystitis with three of the following symptoms or signs

• Abdominal pain in the upper right quadrant

• Murphy's sign

• Leukocytosis > 10 × 10^3^/μl

• Rectal temperature > 38°C or < 36.5°C

plus

• Cholecystolithiasis (stones/sludge) or sonographic signs of cholecystitis (thickening and triple layer formation of the gall bladder wall)

• Immediate antibiotic therapy (400 mg Moxifloxacin i.v. once a day)

• Laparoscopic cholecystectomy possible within 24 hours after presentation of the patient

• Informed consent

### Exclusion criteria

• ASA IV and V (table 1)

**Table 1:** ASA-Criteria

***ASA Physical Status (PS) Classification System from the American Society of Anesthesiologists***	
***ASA PS Category***	***Preoperative Health Status***

ASA PS 1	Normal healthy patient
ASA PS 2	Patients with mild systemic disease
ASA PS 3	Patients with severe systemic disease
ASA PS 4	Patients with severe systemic disease that is a constant threat to life
ASA PS 5	Moribund patients who are not expected to survive without the operation
ASA PS 6	A declared brain-dead patient who organs are being removed for donor purposes

American Society of Anesthesiologists (ASA) Physical Status (PS) Classification System. Categories to classify the preoperative health status of patients.

• Septic shock

• Perforation or abscess of the gall bladder

• No possibility of laparoscopic surgery

• Additional antibiotics needed for secondary disease

• Intolerance to moxifloxacin or other quinolones

• Pregnancy (also suspected), breast feeding

• Life-expectancy < 48 hours

• End-stage liver disease (Child-Pugh C)

• Psychiatric or severe neurologic disease

• Relevant bradycardia or other symptomatic arrhythmias

• Significant cardiac disease

• Disorder with QT prolongation

• Hypocalcaemia or other electrolyte disorders

• Earlier participation in this trial

## Ethics, Study Registration and Consent

The final protocol was approved by the independent ethics committee of the University of Heidelberg. The study was registered at ClinicalTrials.gov (NCT00447304). Patients who are scheduled for laparoscopic cholecystectomy (immediate or elective) due to acute cholecystitis are informed about the trial (laparoscopic surgery, possibility of conversion to open surgery, other risks, benefits and confidential handling of documented findings) and are given the opportunity to participate at the screening visit. Informed consent is required. Patients may withdraw from the study at any time without giving reasons and without jeopardizing their further treatment. The investigator may also withdraw patients if this is in their best interests.

## Randomisation and procedures for minimizing bias

This study is randomized to minimize bias. Sealed randomization envelopes are provided in packs of four by the CRO and are held centrally at each investigational site. An envelope is opened when a patient agrees to take part and patients are informed whether they are to be treated with immediate surgery or initial conservative antibiotic therapy.

## Study treatment

Day 1 is defined as the day the patient presents to the hospital. He undergoes a physical examination, vital signs are documented, and an abdominal ultrasound investigation is performed to confirm the diagnosis of acute cholecystitis. A blood sample is also taken for standard laboratory diagnosis (including Na, K, INR, Hb, platelets, leukocytes, bilirubin, ALT, AST, gamma-GT, AP, amylase, lipase, urea, creatinin and CRP). All relevant concomitant diseases (e.g. coronary heart disease, diabetes mellitus) indicative for morbidity and mortality are recorded [28].

The patient is informed about the trial and is given the opportunity to participate. After the patient has given his informed consent, he is randomized, and all baseline findings, date of birth, age, sex, medical history, height and weight are documented in the CRF.

All patients are examined daily while in the hospital. The laboratory investigations are repeated on Day 3. Samples are taken for microbiological cultures, if necessary. At Day 75 (test-of-cure visit), all diagnostic procedures and treatments between Day 1 and Day 75 are documented. The laboratory determinations and physical examination are repeated, and vital signs are measured. Morbidity is documented according to Table 2.

**Table 2:** Morbidity Score

Persistent abdominal pain > 72 h	1	Pain treated by morphine or derivatives > 72 h
Persistent fever > 72 h	1	Rectal temperature > 38.5°C at least twice
Persistently raised signs of infection > 72 h	1	Persistently elevated CRP or leukocytosis
Wound-healing disorder	2	Any problem leading to re-opening of the wound with subsequent open wound treatment
Thrombosis	3	New onset of leg or pelvic thrombosis
Bleeding	3	Need for more than two bags of packed red cells during or after surgery
Cholangitis	3	New increase in AP, GGT (>2× ULN), bilirubin (>1× ULN) plus leukocytosis (> 12 × 10^3^/μl) or increase in CRP (> 5× ULN)
Icterus	3	New increase in bilirubin, AP and GGT (>2× ULN)
Bile leakage	3	Persistent leakage shown by CT, MRI or ERCP
Abscess	3	Shown by CT, MRI or ultrasound
Pneumonia	3	Shown by X-ray plus drop in arterial pO_2 _plus clinical signs of pneumonia plus leukocytosis plus increased CRP
Embolic lung disease	4	Increased PA pressure (echocardiogram), TNT/TNI, D-dimers
Peritonitis	4	New occurrence of peritonitis
Pancreatitis	4	Increased pancreatic enzymes (> 3× ULN) plus new increase in CRP (> 5× ULN) plus positive clinical signs
Renal failure	4	Drop in urine production below 500 mL/day plus increased creatinine and urea (> 2× ULN)
Relaparotomy	5	Need for follow-up surgery
Cerebral ischemia or bleeding	5	New neurological symptoms with corresponding to changes in cerebral CT
Myocardial infarction	5	Changes in TNT/TNI with or without changes in the ECG meeting the criteria of STEMI of NSTEMI
Septic shock	5	Leukocytosis (> 12 × 10^3^/μl) or leukopenia (< 4 × 10^3^/μl) plus temperature < 36.5°C or > 38.5°C plus clinical signs
Death	63	(Sum of all complications + 1)

Different complications and side effects that may affect the patients during the study are listed and scored differently in increasing severity. Death as worst outcome is scored the sum of all complications plus 1.

### Procedure for patients receiving immediate surgery

• Laparoscopic cholecystectomy in the 24 hours after hospital admission

• Antibiotic therapy with moxifloxacin 400 mg i.v. once per day for 48 hours followed by oral Moxifloxacin 400 mg daily or discontinuation of antibiotic treatment if possible

• Discharge of the patient as soon as possible after Day 2, if the body temperature, CRP and leukocytes are normal

• Test-of-Cure visit at Day 75

### Procedure for patients receiving primarily conservative therapy with elective surgery

• Therapy with i.v. moxifloxacin 400 mg once daily for 48 hours followed by oral moxifloxacin 400 mg per day. Discontinuation of moxifloxacin after Day 7, provided body temperature, CRP and leukocytes are normal

• Discharge of the patient as soon as possible after Day 4 on oral moxifloxacin

• Elective cholecystectomy between Days 7 and 45 after admission to study using single-shot moxifloxacin i.v. for prophylaxis

• Test-of-Cure visit at Day 75

## Primary and secondary endpoints

### Primary endpoints

Primary endpoint is morbidity at the test-of-cure (TOC) visit (75 days after trial inclusion) in the tested population valid for efficacy.

### Secondary endpoints

1. Morbidity over 75 days using the scoring system showed in Table 2

2. Morbidity 3 days after cholecystectomy (immediate and elective)

3. Rate of conversion from laparoscopic to open surgery

4. Change of antibiotic due to non-response or non-toleration of moxifloxacin

5. Mortality at Day 75

6. Cost-efficiency

7. Hospital time

8. Safety and tolerance of moxifloxacin

9. Duration of hospital stay after cholecystectomy (days)

## Adverse events and serious adverse events

Adverse events and serious adverse events and deaths occurring up to Day 75 were recorded. An adverse event (AE) is every medical event that worsens or impairs the well-being of the patient not being part of the natural course of the disease but may be due to treatment or drug application. The term AE can cover any sign, symptom or reaction, including not normal laboratory findings, independent if caused by the tested procedure and medication or not. Adverse event intensity (mild, moderate or severe) and relationship to the treatment or the study drug moxifloxacin (probable, possible, unlikely or none) were categorized. Serious adverse events (SAEs) included those events that were fatal, life-threatening, required hospitalization, resulted in disability or otherwise endangered the patient.

All AEs and SAEs will be documented in detail in the case report form (CRF) and will be reported to the principal investigator at regular intervals. SAEs have to be reported within 24 hours to the principal investigator, and must be documented separately on an SAE report form within 24 hours. According to law and guidelines, SAEs have to be reported to the ethics committee(s) and supervisory board(s) as necessary. The period of observation for AE reporting is from Day 1 to Day 75.

## Discussion

All statistical tests will be performed two-sided with a level of significance of 5%. The patients will be analyzed as treated. The principle analysis will be on evaluable patients and a supportive intent-to-treat analysis will be performed. Patients will be stratified according to severity of their condition (ASA ≤ 2 vs. ASA > 2); the principle analysis will be stratified, but a non-stratified analysis will also be performed

The groups will be tested for equivalence of distribution of age, sex and body mass index. The analysis will be conducted using analysis of variance with the factors study group and severity of disease, stratified by the Cochran-Mantel-Haenszel test.

95% confidence intervals (CI) for the difference of morbidity rates will be calculated. If and only if this CI lies between -10% and +10% the two procedures will be considered equivalent. The calculation of the 95% CI will be stratified by severity (ASA ≤ 2 vs. ASA > 2). A Breslow-Day test will be performed to check for homogeneity between these two strata.

Amongst the secondary variables, the mean morbidity score in the two groups stratified by severity of disease will be compared using the Wilcoxon rank-sum test. Where appropriate, descriptive statistics will also be performed for all other variables.

## Study organization

All eligible patients are seen by a gastroenterologist or surgeon and are enrolled after giving informed consent. The incidence of patients with acute cholecystitis ranges from 10 to >100 per year at different investigational sites. With about 30 sites, it is estimated that enrollment of 644 patients will take about 24 months. All findings are recorded in the patients medical records and CRF provided for this study by the investigator. Data verification is performed by the CRO, who will also perform the analysis on the locked database after plausibility testing and data query correction.

## Competing interests

The author(s) declare that they have no competing interests.

## Authors' contributions

KW participated in the design and coordination of the study and drafted the manuscript. JE, CG and JK participated in the design and coordination and helped to draft the manuscript. JE and CG conceived the study. JE, CG, JK and KW supervised the coordination of the different trial centers. MWB and WS supervised the coordination of the study. All authors read and approved the final version of the manuscript.
